# Vitamin A deficiency and inflammatory markers among preschool children in the Republic of the Marshall Islands

**DOI:** 10.1186/1475-2891-3-21

**Published:** 2004-12-08

**Authors:** Maria Maqsood, Barbara Dancheck, Mary V Gamble, Neal A Palafox, Michelle O Ricks, Kennar Briand, Richard D Semba

**Affiliations:** 1 Wilmer Eye Institute, Johns Hopkins University School of Medicine, Baltimore, Maryland, USA; 2 Mailman School of Public Health, Columbia University, New York, New York, USA; 3 Department of Family Practice and Community Health, John A. Burns School of Medicine, University of Hawaii, Honolulu, Hawaii, USA; 4 Ministry of Health and Environment, Republic of the Marshall Islands

**Keywords:** acute phase response, inflammation, retinol, vitamin A deficiency, xerophthalmia

## Abstract

**Background:**

The exclusion of individuals with elevated acute phase proteins has been advocated in order to improve prevalence estimates of vitamin A deficiency in surveys, but it is unclear whether this will lead to sampling bias. The purpose of the study was to determine whether the exclusion of individuals with elevated acute phase proteins is associated with sampling bias and to characterize inflammation in children with night blindness.

**Methods:**

In a survey in the Republic of the Marshall Islands involving 281 children, aged 1–5 years, serum retinol, C-reactive protein (CRP), and α_1_-acid glycoprotein (AGP) were measured.

**Results:**

Of 281 children, 24 (8.5%) had night blindness and 165 (58.7%) had serum retinol <0.70 μmol/L. Of 248 children with AGP and CRP measurements, 123 (49.6%) had elevated acute phase proteins (CRP >5 mg/L and/or AGP >1000 mg/L). Among children with and without night blindness, the proportion with serum retinol <0.70 μmol/L was 79.2% and 56.8% (*P *= 0.03) and with anemia was 58.3% and 35.7% (*P *= 0.029), respectively. The proportion of children with serum retinol <0.70 μmol/L was 52.0% after excluding children with elevated acute phase proteins. Among children with and without elevated acute phase proteins, mean age was 2.8 vs 3.2 years (*P *= 0.016), the proportion of boys was 43.1% vs. 54.3% (*P *= 0.075), with no hospitalizations in the last year was 11.0% vs 23.6% (*P *= 0.024), and with anemia was 43.8% vs 31.7% (*P *= 0.05), respectively.

**Conclusions:**

Exclusion of children with inflammation in this survey of vitamin A deficiency does not improve prevalence estimates for vitamin A deficiency and instead leads to sampling bias for variables such as age, gender, anemia, and hospitalization history.

## 

Vitamin A deficiency is a major cause of morbidity and mortality among preschool children in developing countries [1]. Vitamin A, or all-*trans *retinol, is available as preformed vitamin A in foods such as eggs and dairy products and as provitamin A carotenoids in foods such as dark green leafy vegetables, pumpkin, and papaya. Vitamin A is essential for normal immune function, hematopoiesis, growth, and vision [1]. Among preschool children, risk factors for vitamin A deficiency include age, such as the period that follows weaning when vitamin A intake is low and risk of precipitating infections is high [2,3], recent infections such as diarrheal disease [4], and low socioeconomic status [5]. The syndrome of vitamin A deficiency is characterized by increased susceptibility to infections, anemia, and elevated acute phase proteins [1,6,7].

The identification of populations at risk for vitamin A deficiency is important in order to target groups that would benefit from interventions to improve vitamin A status. Xerophthalmia, such as night blindness and Bitot spots, is used to determine the prevalence of clinical vitamin A deficiency in populations [1]. Other surveys that are conducted to determine whether vitamin A deficiency is a public health problem often rely upon the measurement of serum or plasma retinol concentrations [8]. However, plasma retinol is a negative acute phase reactant that decreases during infections [9], and it has been thought that the measurement of plasma retinol concentrations may not accurately reflect the vitamin A status of populations with a high prevalence of subclinical infections, as some portion of low retinol concentrations may be due to an acute phase response [10]. Recently, Thurnham and colleagues proposed that in surveys that rely upon plasma or serum retinol concentrations to estimate the prevalence of vitamin A deficiency, one method to improve the accuracy of the prevalence estimates is to exclude all individuals with elevated acute phase proteins [11]. The underlying assumption using this method is that subclinical infection is randomly distributed in a population, and that the exclusion of individuals with elevated acute phase proteins will not lead to sampling bias.

We hypothesized that exclusion of individuals with elevated acute phase proteins, and selection of only apparently healthy children would lead to lead to sampling bias in a survey of vitamin A deficiency. A secondary hypothesis was that children with night blindness would have elevated acute phase proteins. Vitamin A deficiency has been recognized as a major public health problem in many areas of the South Pacific region [12], including the Republic of the Marshall Islands [13,14]. In order to address these hypotheses, we characterized serum retinol concentrations, night blindness, and markers of inflammation in a population-based survey among preschool children in the Republic of the Marshall Islands.

## Subjects and Methods

A population-based survey, the Republic of the Marshall Islands Vitamin A Deficiency Study, was conducted between November 1994 and March 1995 to assess the prevalence of vitamin A deficiency among children, aged 1–5, in the Republic of the Marshall Islands. The Republic of the Marshall Islands (population 68,000) is a complex of 28 coral atolls and 5 small (non-atoll) islands, with a total of 1136 islands spreading over a very small land area of 134 km^2^. The sampling strategy for the study was based on the 1988 census of the Republic of the Marshall Islands, which provided data on the average number of children of the target age group within each household, determined by dividing the number of children in a locality by the number of households in the same location. This number was then divided into the number of children to be sampled to obtain the number of households to be visited. Households to be visited were chosen by systematic sampling of every fifth household. When available, the birth dates of the children were ascertained from the children's health cards; otherwise, the birth dates were obtained by asking the parent or guardian. The survey teams consisted of at least one Marshallese-speaking health care worker, a phlebotomist, and a medical doctor.

Of the children in the survey, a systematic subsample of every fifth child was obtained in which a standardized questionnaire was used to collect information on various risk factors such as night blindness, demographics, breastfeeding, visits to the hospital, presence of a vegetable garden, and food consumption. Parents were asked if the child currently had night blindness. A history of night blindness has been shown to be a reliable indicator of vitamin A deficiency [15]. Oral informed consent was obtained from a parent or guardian prior to participation in the survey as considered appropriate by the institutional review board for this setting.

Blood samples were obtained by venipuncture. Hemoglobin was measured using a HemoCue instrument (HemoCue, Inc., Mission Viejo, CA). Venous blood samples were collected in red-top serum separator tubes and immediately wrapped in aluminum foil, continuously shielded from light, and stored at 4°C until centrifugation (200 *g *× 10 min, room temperature). Aliquots of serum were made, kept in liquid nitrogen or at -70°C, and shipped by overnight air express from the Republic of the Marshall Islands to New York City. Retinol remains stable at -70°C for 15 y or more [16]. Serum retinol was measured using reverse-phase high performance liquid chromatography, as described elsewhere [13]. Serum C-reactive protein (CRP) was determined using a commercial enzyme-linked immunosorbent assay kit (Hemagen Diagnostics, Waltham, MA). Serum α_1_-acid glycoprotein (AGP) was measured using radial immunodiffusion assay (Bindarid, The Binding Site, Birmingham, UK). Interleukin-6 (IL-6) was analyzed using a high sensitivity commercial enzyme-linked immunosorbent assay (R&D systems, Inc, Minneapolis, MN). Serum retinol, CRP, AGP, and IL-6 values were available for 281, 250, 248, and 176 of 281 pre-school children, respectively. Missing values were due to inadequate volume of sera. Controls provided by the manufacturer were used to measure intra- and inter-assay coefficients of variation (CV) in laboratory analysis. For serum retinol, the within-assay and between-assay CVs were 3% and 8%, respectively. For serum CRP, AGP, and IL-6, the within-assay and between-assay CVs were 4.1 and 5.0, 3.9 and 1.6, and 3.2 and 2.9, respectively. The study protocol was approved by the institutional review board of the Pacific Health Research Institute of Hawaii and the Ministry of Health and Environment of the Republic of the Marshall Islands. The study was conducted in accordance with the Helsinki Declaration.

Groups were compared using Student's *t*-test or analysis of variance for continuous variables where appropriate, and categorical variables were compared using χ^2 ^or exact tests. Vitamin A deficiency was defined as serum retinol <0.70 μmol/L [1]. Inflammation was defined as CRP >5 mg/L and/or AGP >1000 mg/L [6,7,11]. Anemia was defined as hemoglobin <110 g/L [14]. Nonparametric Wilcoxon rank sum test was used on continuous variables in which the distribution was not normal. The proportion of children with serum retinol <0.70 μmol/L was "adjusted" using two methods: (1) excluding all children with inflammation and then presenting the proportion of children who had serum retinol <0.70 μmol/L for the remaining children without inflammation, and (2) calculating geometric mean serum retinol concentrations in children with and without inflammation, multiplying the serum retinol concentrations in the group with inflammation by a constant that makes the geometric mean serum retinol in the group with inflammation equivalent to the group without inflammation, and then reporting the proportion of children who had serum retinol <0.70 μmol/L [11]. One sample test of proportions was used to compare proportions of children with serum retinol <0.70 μmol/L between "adjusted" and unadjusted groups. Spearman correlation was used to examine correlation between AGP, CRP, IL-6, retinol, and hemoglobin. The level of significance used in this study was *P *< 0.05.

## Results

A total of 919 Marshallese children, age 1–5, participated in the survey, and the systematic subsample of every fifth child involved 281 children from the following atolls (n): Ailuk (16), Arno (26), Enejelar (3), Enewetak (14), Kwajalein (73), Majuro (77), Namu (32), Utrik (25), and Wotje (15). There were 141 boys and 140 girls, and the mean (±standard deviation) age of the children was 2.93 ± 1.38 years. Due to limited sample volume, serum CRP, AGP, and IL-6 were not measured in 31, 33, and 105 pre-school children, respectively. Of the 281 children, there were 24 (8.5%) with night blindness and 165 (58.7%) with serum retinol <0.70 μmol/L. Of 248 children who had both AGP and CRP measured, there were 123 (49.6)% with inflammation (CRP >5 mg/L and/or AGP >1000 mg/L).

The characteristics of children with and without inflammation are shown in Table 1. Children with inflammation were older (*P *= 0.016), were more likely to have been hospitalized in the last year (*P *= 0.024), and were more likely to be anemic (*P *= 0.05) compared with children without inflammation. Mean retinol concentration was lower (*P *= 0.0005) and the proportion of children with serum retinol <0.70 μmol/L was higher (*P *= 0.013) among children with inflammation compared to children without inflammation. The findings suggest that children with inflammation were more likely to be girls (*P *= 0.075) compared to children without inflammation. There were no significant differences in the proportion with night blindness, the mean number of people living in the house with the child, current breastfeeding status, and the presence of a vegetable garden at home between children with and without inflammation.

**Table 1:** Relationship between inflammation and demographic characteristics of children, age 1–5 years, in the Republic of the Marshall Islands

	**Inflammation ^1^**		
**Characteristic ^2^**	**No (*n *= 125)**	**Yes (*n *= 123)**	***P***
Mean age (years)	3.2 (3.0, 3.4)	2.8 (2.5, 3.0)	0.016
Sex (% male)	54.3	43.1	0.075
Night blind (%)	9.5	8.9	0.83
Hospitalized in last year (%)			
0	23.6	11.0	0.024
1–2	44.7	43.2	
3–4	18.7	31.4	
5+	13.0	14.4	
Mean number of people living in house	11.4 (10.1, 12.7)	11.3 (10.1, 12.4)	0.62
Currently breastfeeding (%)	14.3	18.9	0.33
Presence of a vegetable garden at home (%)	32.2	32.8	0.93
Mean hemoglobin (g/L)	119 (108, 112)	109 (106, 110)	0.19
Hemoglobin <110 g/L (%)	31.7	43.8	0.05
Mean retinol (μmol/L)	0.66 (0.61, 0.72)	0.52 (0.47, 0.58)	0.0005
Retinol <0.70 μmol/L (%)	52.0	67.5	0.013

^1 ^Defined as CRP >5 mg/L and/or AGP >1000 mg/L. ^2 ^Geometric mean; 95% CI in parentheses.

Of the 281 children 58.7% had serum retinol <0.70 μmol/L. In contrast, if children with inflammation are excluded as proposed by Thurnham and colleagues [11], the proportion with serum retinol <0.70 μmol/L is 52.0% (Table 1). Alternatively, it has been proposed that serum retinol concentrations are "adjusted using acute phase proteins" based upon retinol values among children with and without inflammation [11]. When this alternative method is used, the proportion of children with serum retinol <0.70 μmol/L is 47% (95% C.I. 0.43–0.60), compared with the unadjusted proportion of 58.7% (*P *= 0.08).

The characteristics of children with and without night blindness are shown in Table 2. Children with night blindness were slightly older (*P *= 0.03), had lower serum retinol (*P *= 0.007), were more likely to have serum retinol <0.70 μmol/L (*P *= 0.03), and to be anemic (*P *= 0.029) compared to children without night blindness. The findings were suggestive that there may be a higher proportion of boys than girls who are night blind (*P *= 0.09), and with lower mean hemoglobin concentrations (*P *= 0.09). There were no significant differences in geometric mean AGP, CRP, IL-6, or inflammation between children with and without night blindness.

**Table 2:** Characteristics of children, aged 1–5 years, with and without night blindness in the Republic of the Marshall Islands

	**Night blindness**		
**Characteristic ^1^**	**Yes (*n *= 24)**	**No (*n *= 257)**	***P***
Mean age (years)	3.5 (3.0, 4.0)	2.9 (2.7, 3.1)	0.03
Sex (% male)	66.7	48.6	0.09
Geometric mean AGP (mg/L)	925 (819, 1044)	924 (881, 970)	0.99
AGP >1000 mg/L (%)	43.5	45.8	0.83
Geometric mean CRP (mg/L)	1.3 (0.2, 11.8)	1.7 (0.1, 324.3)	0.36
CRP >5 mg/L (%)	8.7	23.1	0.11
With inflammation (%) ^2^	47.8	49.3	0.89
Geometric mean IL-6 (pg/mL)	3.9 (0.9, 14.1)	3.9 (0.7, 16.2)	0.96
Mean retinol (μmol/L)	0.40 (0.04, 1.00)	0.62 (0.02, 1.90)	0.007
Retinol <0.70 μmol/L (%)	79.2	56.8	0.03
Mean hemoglobin (g/L)	106 (101, 110)	109 (108, 110)	0.09
Hemoglobin <110 g/L (%)	58.3	35.7	0.029

^1 ^For continuous variables (95% CI). ^2 ^Inflammation defined as AGP >1000 mg/L and/or CRP >5 mg/L.

Spearman correlations of serum AGP, CRP, IL-6, retinol, and hemoglobin are shown in Table 3. Serum AGP was positively correlated with serum IL-6 and CRP (*P *< 0.0001 for both). Serum AGP and CRP individually were inversely correlated with serum hemoglobin (*P *< 0.0001) and retinol (*P *= 0.0004). IL-6 was positively associated with CRP (*P *< 0.0001), and inversely associated with retinol (*P *< 0.0001). Hemoglobin had low correlation with retinol that was of borderline significance (*P *= 0.09) and low inverse correlation with IL-6 that was also of borderline significance (*P *= 0.08).

**Table 3:** Spearman correlation between AGP, CRP, IL-6, retinol, and hemoglobin in preschool children in the Republic of the Marshall Islands

	**Hemoglobin**	**Retinol**	**IL-6**	**CRP**
**AGP**	-0.19	-0.25	0.48	0.60
	*P *= 0.0026	*P *< 0.0001	*P *< 0.0001	*P *< 0.0001
**CRP**	-0.13	-0.22	0.44	
	*P *< 0.0001	*P *= 0.0004	*P *< 0.0001	
**IL-6**	-0.12	-0.28		
	*P *= 0.08	*P *< 0.0001		
**Retinol**	0.10			
	*P *= 0.09			

## Discussion

In this population of children from the Republic of the Marshall Islands, more than half had serum retinol concentrations <0.70 μmol/L. According to criteria of the World Health Organization, vitamin A deficiency is considered a public health problem if more than 15% of the population has serum or plasma retinol concentrations <0.70 μmol/L [17]. Vitamin A deficiency is also considered a public health problem if >1% of children less than six years old have night blindness [1], and in this survey, the prevalence of night blindness was more than eight times higher than this criterion. Thus, vitamin A deficiency was certainly a public health problem among children, aged 1–5 years, in the Republic of the Marshall Islands. Since the time of this survey, the Republic of the Marshall Islands has implemented a countrywide vitamin A capsule distribution program.

Nearly half of the children in this study had inflammation, as indicated by elevated AGP and/or CRP. These findings suggest that the prevalence of subclinical infection is high in this population that has a high prevalence of vitamin A deficiency. These findings are consistent with the concept that the syndrome of vitamin A deficiency is associated with depressed immunity and increased infections [6,7]. Children with subclinical vitamin A deficiency may have pathological alterations in T and B cell function and mucosal immunity that make them more susceptible to subclinical infections, such as diarrheal disease [18].

Although the exclusion of individuals with elevated acute phase proteins has been advocated to improve prevalence of vitamin A deficiency in surveys that rely upon plasma or serum retinol concentrations [11], this study shows that exclusion of these individuals leads to sampling bias. The remaining subjects in the sample without inflammation are different from those excluded from the sample of the study, as there was selection bias in regard to age, gender, anemia, and morbidity history. Thus, by excluding those with inflammation, the prevalence estimates of vitamin A deficiency are based upon a biased sample that may be healthier. Subclinical infections are more likely to occur among malnourished children and among children from poorer families [19,20]. Studies among adults suggest that elevated acute phase proteins are more common among those with lower socioeconomic status [21,22]. The present study is limited in that data on maternal education, socioeconomic status, and other demographic indicators was not collected. Similar analyses could be conducted with other large existing data sets to corroborate and characterize the extent of sampling bias in vitamin A surveys when individuals with elevated acute phase proteins are excluded.

The alternative method of having serum retinol concentrations "adjusted using acute phase proteins" [11] involves the same problem of sampling bias, as the group without inflammation that is used for "adjusting" the serum retinol concentrations of the group with inflammation is different as discussed above.

Children with night blindness had significantly lower serum retinol concentrations compared with children without night blindness, a finding that is consistent with previous studies [6,7,15]. The ability to see at night depends on the visual pigment, rhodopsin, in rod photoreceptors of the retina. Synthesis of rhodopsin depends in part upon the availability of circulating retinol. The prevalence of anemia was higher among children with night blindness than children without night blindness. Vitamin A deficiency is associated with anemia, and there may be several mechanisms by which vitamin A deficiency could cause anemia, including impairment of iron metabolism, and immune dysfunction and associated anemia of infection [23]. In the present study, children with night blindness were not more likely to have elevated acute phase proteins than children without night blindness. However, the number of children with night blindness in this study was small, and the study had limited statistical power to address this secondary hypothesis. Other studies among pregnant women in Nepal [6] and preschool children in Indonesia [7] show that individuals with night blindness are more likely to have elevated acute phase proteins.

The relationship between IL-6 and elevated acute phase proteins has not well been characterized in epidemiological studies of vitamin A deficiency. IL-6 is a proinflammatory cytokine that plays a role in the upregulation of CRP [24] and AGP [25]. In the present study, IL-6 concentrations had a moderate correlation with both CRP and AGP. CRP is one component of a first line of innate host defense against infectious diseases [24]. The biological function of AGP has not been well characterized [25]. The inverse correlation of hemoglobin with AGP, CRP, and IL-6 suggests the role of proinflammatory cytokines and inflammation in the anemia of chronic infection [26].

In summary, the method of excluding individuals with elevated acute phase proteins from this survey of vitamin A deficiency resulted in sampling bias and a prevalence estimate that was based upon about half of the original sample. The remaining sample was healthier, older, less likely to have been hospitalized, and with a higher proportion of boys and a lower proportion with anemia. The method of excluding individuals with elevated acute phase proteins may lead to underestimation of the prevalence of vitamin A deficiency. Vitamin A deficiency remains a major problem in many developing countries worldwide, and further studies are needed to develop unbiased epidemiological methods for the estimation of the prevalence of vitamin A deficiency in populations.
